# Predictors of consistent condom use based on the Information-Motivation-Behavior Skill (IMB) model among senior high school students in three coastal cities in China

**DOI:** 10.1186/1471-2334-13-262

**Published:** 2013-06-04

**Authors:** Yong Cai, Xiuxia Ye, Rong Shi, Gang Xu, Lixiao Shen, Jia Ren, Hong Huang

**Affiliations:** 1 School of Public Health affiliated with School of Medicine, Shanghai Jiaotong University, No.227, South Chongqing Road, Shanghai 200025, PR China; 2 MOE - Shanghai Key Laboratory of Children’s Environmental Health, Xinhua Hospital, affiliated with School of Medicine, Shanghai Jiaotong University, No.1665, Kongjiang Road, Shanghai 200092, PR China; 3 Shanghai Children’s Medical Center, affiliated with School of Medicine, Shanghai Jiaotong University, No.1678, Dongfang Road, Shanghai 200127, PR China

**Keywords:** Senior high school students, IMB model, Consistent condom use

## Abstract

**Background:**

High prevalence of risky sexual behaviors and lack of information, skills and preventive support mean that, adolescents face high risks of HIV/AIDS. This study applied the information-motivation-behavioral skills (IMB) model to examine the predictors of consistent condom use among senior high school students from three coastal cities in China and clarify the relationships between the model constructs.

**Methods:**

A cross-sectional study was conducted to assess HIV/AIDS related information, motivation, behavioral skills and preventive behaviors among senior high school students in three coastal cities in China. Structural equation modelling (SEM) was used to assess the IMB model.

**Results:**

Of the 12313 participants, 4.5% (95% CI: 4.2–5.0) reported having had premarital sex and among them 25.0% (95% CI: 21.2–29.1) reported having used a condom in their sexual debut. Only about one–ninth of participants reported consistent condom use. The final IMB model provided acceptable fit to the data (CFI = 0.981, RMSEA = 0.014). Consistent condom use was significantly predicted by motivation (β = 0.175, *P* < 0.01) and behavioral skills (β = 0.778, *P* < 0.01). Information indirectly predicted consistent condom use, and was mediated by behavioral skills (β = 0.269, *P* < 0.05).

**Conclusions:**

The results highlight the importance of conducting HIV/AIDS preventive health promotion among senior high school students in China. The IMB model could predict consistent condom use and suggests that future interventions should focus on improving motivation and behavioral skills.

## Background

HIV/AIDS is a global health issue and public health challenge. There are an estimated 33 million people living with HIV globally, half of whom are female [1]. In the past decade, China has experienced a rapid increase in HIV/AIDS cases. As of the end of 2011, it is estimated that there were 780,000 (620,000–940,000) people living with HIV/AIDS (PLHIV) in China, among whom 46.5% contracted HIV through heterosexual contact and 17.4% through homosexual contact [2]. New cases of HIV infection in China numbered about 48,000 in 2011, and sexual contact remains the major route of HIV transmission and also continues to grow [3]. Sexual transmission (heterosexual and homosexual) accounted for 81.6% of new HIV infection cases diagnosed in China in 2011 [2]. Therefore, effective control of sexual transmission through advocating safe sex to reduce risky sexual behavior will significantly contribute to HIV/AIDS prevention, especially among sexually active young people [4,5]. Young people account for a significant proportion of new HIV infections. For example, by using improved methodology, it estimated HIV incidence among persons aged 13 years and older, 13–29 year old age group made up 32%, 36%, 39%, and 39% of new infections during 2006 to 2009 in the United States [6].

Adolescents are often characterized as being at a life stage of increased experimentation and exploration associated with a range of risky behaviors, including risky sexual behaviors [7]. Moreover, adolescents are less likely than adults to have the information, skills and support to protect themselves against HIV/AIDS [8]. A recent report on risky behaviors among American adolescents aged 10–24 years old indicated that 47.8% had engaged in sexual intercourse, and 38.5% of those who were sexually active had engaged in sexual intercourse without a condom [9]. In many developing countries, adolescents have become increasingly likely to engage in habitual risky sexual behavior such as early sexual initiation and unprotected sex [10-12]. As the largest low or middle income nation, China has a population of over 1.3 billion. Of this 1.3 billion, 24.25% are adolescents and youths aged 14 to 29 years old, according to a 2009 report from the National Bureau of Statistics of the People’s Republic of China. With the rapid progress of globalization, economic development and changes in social culture, more Chinese adolescents are becoming sexually active prior to marriage, and are initiating sexual activity earlier than ever [13]. A recent study that surveyed 109,754 students in grades 10–12 and 33,653 college students found that over 4.8% of senior high school students and 11.3% of college students reported having experienced sexual intercourse, while their rate of condom use was below 30%, making them extremely vulnerable to HIV/AIDS and pregnancy [14]. To assist efforts to promote condom use and control the transmission of HIV, a better understanding of risky sexual behaviors and predictors of condom use among adolescents is required. However, information about the predictors of condom use among young Chinese people, especially senior high school students, is very limited because traditional Chinese culture typically regards sex as obscene and immoral [15].

Many studies demonstrate that most Chinese youths lack basic information on sex and HIV/AIDS [16-18]. Few studies have focused on HIV/AIDS related behaviors by using health promotion theoretical frameworks in China [19], and no study has applied the information-motivation-behavioral skills (IMB) model to condom use among adolescents. The IMB model developed by Fisher and his colleagues has shown that HIV prevention information, motivation, and behavioral skills are the fundamental determinants of HIV preventive behavior [20]. The model assumes that an individual must be well-informed, motivated, and possess the necessary self-efficacy behavioral skills to initiate preventive behavior [21]. Tests of the IMB model in studies predicting condom use among at-risk populations have shown it to have good fitness [22-24]. The IMB model provides a valid framework for public health education and prevention. Assuming HIV/AIDS prevention information and motivation affect condom use largely through behavioral skills, while information and motivation may also directly affect condom use, we tested associations among IMB constructs as predictors of condom use by performing structural equation modelling (SEM) on senior high school students from three cities in China.

## Method

### Study site

The study was conducted in three coastal cities in China, namely Shanghai, Beihai in Guangxi Province and Sanming in Fujian Province from year 2007 to 2008. These three coastal cities were selected as study sites because each had a different prevalence of AIDS relative to the rest of China: high in Guangxi, average in Shanghai and low in Fujian.

### Study population and sampling size

We conducted a two-stage cluster sample design by administering a questionnaire on AIDS health awareness to senior high school students from three coastal cities to survey HIV/AIDS related information, motivation, behavioral skills and sexual behaviors from three coastal cities. This self-administered, school-based instrument had been tested in preliminary research and showed good fitness based on reliability and validity analysis [25]. Assuming a prevalence of premarital sexual behaviors of 4.8% [14] among senior high school students, an α of 0.05, and a relative sampling error of 0.1P, we calculated a required sample size of approximately 13,000 to allow for the larger sampling error of the complex samples procedure and an non-response rate of 10%. Representative students were sampled using a two-stage cluster sample design. In the first stage, two districts from each city were randomly selected and the sampling frame included all schools in the six selected districts. Forty-nine senior high schools (including technical schools) were then randomly chosen from the six selected districts. In the second stage, six classes within each selected school in the 10th and 11th grade were randomly selected and all students in each sampled class participated in the survey. Twelfth-grade students were preparing for the national college entrance examination at the time of sampling and so were excluded from the sampling frame. All students in attendance on the day the survey was administered were eligible to participate. The sample size was 13,000 students, from whom 12,313 useable questionnaires were collected, for a response rate of 94.7%.

### Ethics

The study was reviewed and approved by the Ethics Committee of the School of Public Health, Shanghai Jiaotong University. Before enrolment in the study, all students and their parents or guardians provided written informed consent, which included the study objectives and procedures, and potential risks and benefits of participation.

### Data collection

Data were collected by self-administered questionnaires which had been tested in preliminary research and showed suitability based on reliability and validity analysis [14]. Prior to participation, we explained the survey aims and general content to each subject and emphasized that participation was voluntary and anonymous. The procedure lasted approximately 20 minutes and every student completed the questionnaire independently. The questionnaires included demographic information, such as age, gender, hometown, family monthly income, hometown, school type (model senior high school, common senior high school or vocation high school) and the constructs of the IMB model (Additional file 1). The IMB model constructs included HIV/AIDS related information, motivation, behavioral skills, and sexual behaviors. The measures of the IMB model constructs are described below.

### Measures

The IMB model is a causal model with latent variables that mixes of path analysis and confirmatory factor analysis, and has been called a hybrid model. The latent variables include information, motivation and behavioral skills which are hypothesized to reflect key constructs of the IMB model [24]. Preventive behavior is the main outcome and the dependent variable in the model. Each latent variable has several associated observable variables that can be directly measured. Structural equation modelling (SEM) is used to estimate the structural coefficients between constructs or latent variables. Each measure of the IMB model construct is described below.

#### Information

HIV/AIDS related information was measured using 18 items with “yes”, “no” or “do not know” response. Positive answers were assigned a score of one, while negative answers or responses of “do not know” received a score of zero. One indicator containing six items was related to safe sex information (e.g., “Do you think the most risky period for conception is about 3 days before menstruation?”; “Do you think condom use can prevent pregnancy?”). Individual question scores were summed and converted into a total score to represent knowledge of safe sex (Cronbach’s alpha coefficient =0.751; range 0–6). The other indicator included 12 items and was related to HIV/AIDS information (e.g., “Do you think people with multiple sexual partners are more likely to catch HIV/AIDS?”; “Do you think HIV/AIDS can be transmitted from a mother to her child during pregnancy or childbirth?”). Individual item scores were summed to produce a total score to represent HIV/AIDS information (Cronbach’s alpha coefficient = 0.750; range 0–12). Higher scores indicate students have access to more information.

#### Motivation

The motivation to take preventative actions against HIV/AIDS was assumed to be a function of individual attitudes or relevant subjective norms [24], and was measured by three indexes constructed from answers on a five-point Likert scale (1 = strongly disagree, 5 = strongly agree). The first index was perceived risk of HIV/AIDS, which contains two items (“Do you agree that you are more likely to get HIV/AIDS if you have no knowledge of this disease?”; “Do you agree that the HIV/AIDS epidemic is rapidly growing?”). The sum of the scores of these two items was taken as the index of susceptibility to HIV/AIDS (Cronbach’s alpha coefficient = 0.608; range 2–10). A high score indicated stronger feeling of vulnerability or susceptibility to HIV/AIDS. The second index was the attitude to condom use and contained four items (e.g., “Do you agree that condoms should be used when having sex?”; “Do you agree that sex without a condom should be refused?”). The sum of the scores of these four items served as the index of attitude to condom use (Cronbach’s alpha coefficient = 0.706; range 4–20), and higher score indicated positive attitude. The third index was of sexual norms and included four items (e.g., “Do you agree with a girl having premarital sex with her boyfriend?”; “Do you agree with extramarital affairs?”). The sum of the scores of these four items served as the index of sexual norms (Cronbach’s alpha coefficient = 0.830; rang 4–20). Higher score indicated a free attitude towards sexual matters.

#### Behavioral skills

Respondent perceptions of their self-efficacy to perform HIV/AIDS preventive behaviors were assessed using two scales. Both scales were constructed from answers on a four-point Liket scale (1 = strongly disagree, 4 = strongly agree).The first scale was condom self-efficacy and consisted of two items (“I can use a condom during sexual intercourse”; “I can persuade my partner to use a condom during sexual intercourse”). The sum of the scores of these two items was converted into the scale of self-efficacy for condom use (Cronbach’s alpha coefficient = 0.916; range 2–8). The second scale was the Rosenberg’s self-esteem scale, containing ten items (e.g., “On the whole, I am satisfied with myself”; “I am able to do things as well as most other people”). The score of these ten items were summed and converted into the scale of self-esteem (Cronbach’s alpha coefficient = 0.730; range 10–40). Higher score indicated more self-efficacy to apply HIV/AIDS preventive skills.

#### Prevention behaviors

Literature review showed that consistent condom use usually indicated HIV/AIDS prevention behavior measures associated with the IMB model [23,24,26]. Consistent condom use was assessed by the item, “Did you use condom consistently during your sexual intercourse in the past 6 months?” Responses were given on a five-point scale (1 = never, 5 = always). A higher score indicated stronger committed to HIV/AIDS prevention behaviors.

### Statistical analysis

Statistical analyses were performed using the Complex Samples Procedure from Statistical Package for Social Sciences (SPSS vision 20.0) for Windows. A weighting factor was applied to each student record to adjust for variation in selection probability at the school and class levels and for nonresponse (by school, class and student). Using the Complex Samples Procedure, we calculated the percentages [95% confidence interval (CI)], means (95% CI) and standard deviation (SD). The structural equation model (SEM) was used to examine the hypothetical IMB model using Amose 20.0. Model fit was assessed using the comparative fit index (CFI), the root mean square error of approximation (RMSEA), and the maximum likelihood chi-square values/degrees of freedom ratio [27]. The CFI compares the proportional improvement in the model relative with a null model, and values greater than 0.9 indicate a good fit. The RMSEA value accounts for model complexity, and value lower than 0.05 indicates a good fit [28]. A non-significant likelihood ratio chi-square test suggests the good model fit, but chi-square is sensitive to sample size, therefore, a *χ*^2^/*df* ratio of 3 or less indicates acceptable fit [27,28]. Confirmatory factor analysis (CFA) was conducted to examine the factor structure (measurement model) and the relationships among all the latent variables and manifest variables [24]. A path model was used to examine predictors of condom use among senior high school students based on the IMB model.

## Results

### Characteristic of participants

A total of 12,313 senior high school students completed the questionnaire (Table 1). Participants had an average age of 17.3 years (95% CI: 17.21–17.33; SD = 0.79; range: 14.9–19.6), and the majority were aged from 17 to 18 years old (N = 6695, 55.4%, 95% CI: 51.0–59.6). Among all participants, 51.6% (N = 6355, 95% CI: 50.6–52.6) were female. About half of the students came from Shanghai (N = 6290, 49.8%, 95% CI: 46.8–52.9). Most participants (N = 8053, 64.0%, 95% CI: 60.5–67.4) were common senior high school students and 42.5% (N = 5281, 95% CI: 41.4–43.6) earned average personal monthly income of 1,500-2,500 Chinese Yuan (1 USD = 6.30 CNY). Less than 5% of participants reported having engaged in premarital sex (N = 552, 4.5%, 95% CI: 4.1–5.0) and 25.0% of those who were sexually active (N = 132, 95% CI: 21.2–29.1) reported having used a condom in their sexual debut. However, only about one –ninth of participants reported consistent condom use (N = 65, 11.6%, 95% CI: 9.1–14.7).

**Table 1:** **Participant characteristics using the complex samples procedure** (**N** = **12313**)

**Characteristic variables**	**Number**	**Weighting %**	**(95% CI)**
Gender			
Male	958	48.4	(47.4–49.4)
Female	6355	51.6	(50.6–52.6)
Age (years)			
<17	3649	29.3	(25.7–33.2)
17–18	6695	55.4	(51.0–59.6)
>18	1969	15.3	(12.4–18.7)
Hometown			
Shanghai	6290	49.8	(46.8–52.9)
Sanming	3027	24.3	(21.4–27.5)
Beihai	2996	25.9	(23.2–28.7)
School type			
Model senior high school	2371	19.0	(16.0–22.5)
Common senior high school	8053	64.0	(60.5–67.4)
Vocational high school	1889	16.9	(14.6–19.6)
Average monthly income per person (CNY*)			
<1500	3982	32.7	(31.3–34.1)
1500-2500	5281	42.5	(41.4–43.6)
>2500	3050	24.8	(23.7–25.9)
Experience of premarital sex			
Yes	552	4.5	(4.1–5.5)
No	11761	95.4	(95.0–95.9)
Condom use in sexual debut			
Yes	132	25.0	(21.2–29.1)
No	420	75.0	(70.9–78.8)
Consistent condom use			
Yes	65	11.6	(9.1–14.7)
No	487	89.5	(85.0–90.3)

* *CNY* Chinese Yuan; 6.30 CNY = 1 USD.

### Confirmatory factor analysis

We applied a preliminary confirmatory factor analysis (CFA) model to the 552 participants that reported having had premarital sex to estimate the factor structure and relationships of the all the IMB variables. Table 2 lists the means, standard deviations, ranges, and factor loadings for all scales of the IMB model. All factor loadings in the model were significant (p < 0.05). Students showed low knowledge of safe sex information: the average knowledge test score was 3.38 correct responses out of a possible 6, for a correct response percentage of just 56.3% (3.38/6). For HIV/AIDS information, the mean score was 7.03 correct responses out of a possible 12, for a correct response percentage of just 58.6% (7.03/12).

**Table 2:** **Summary statistics and factor loadings of the IMB model based on confirmatory factor analyses** (**n** = **552**)

**Scales**	**Mean (95% CI)**	SD*	**FL**
Information			
Safe sex information (SSI, Range: 0–6)	3.38 (3.26–3.49)	1.40	0.53
HIV/AIDS information (HAI, Range: 0–12)	7.03 (6.84–7.23)	2.29	0.62
Motivation			
Perceived susceptibility (PS, Range: 2–10)	6.21 (5.97–6.46)	2.91	0.74
Attitude to condom usage (ACU, Range: 4–20)	14.40 (14.03–14.78)	4.50	0.77
Sexual norms (SN, Range: 4–20)	12.25 (11.84–12.66)	4.92	0.63
Behavioral Skills			
Condom self-efficacy (CSE, Range: 2–8)	5.35 (5.17–5.52)	2.09	0.65
Self-esteem (SE, Range: 10–40 )	30.30 (29.72–30.89)	6.97	0.76
Prevention behaviors			
Consistent condom use (CCU, Range: 1–5)	3.10 (2.99–3.21)	1.31	-

* *SD* Standard deviation, *FL* factor loadings, all significant < 0.01.

**Figure 1:**
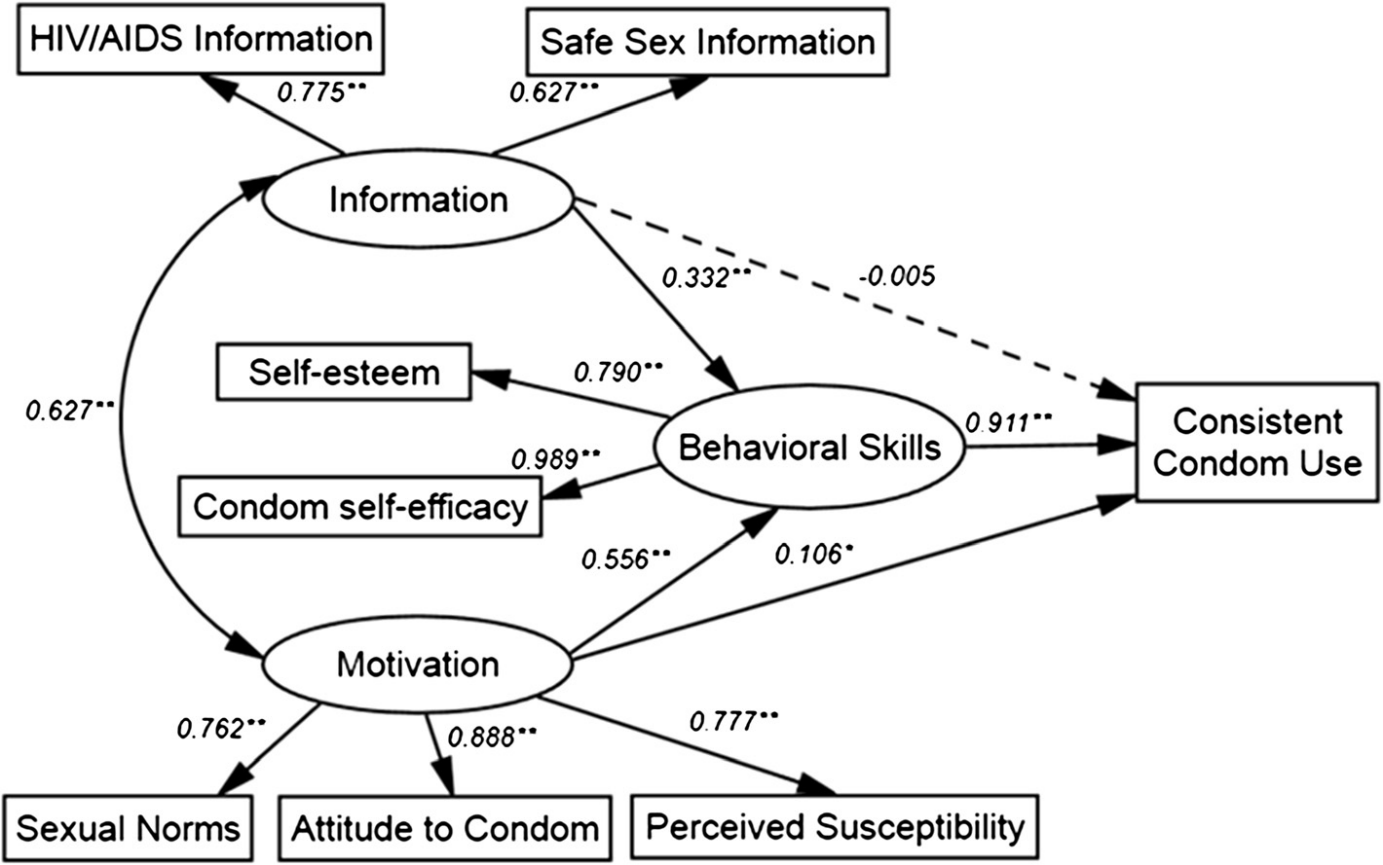
**The preliminary IMB model; predicting condom use among 552 senior high school students.** Oval represent multiple- indicator latent variables, rectangle represent single- indicator observable variables. Single- headed arrow represent regression path, double- headed arrows represent correlations. Dotted line indicates non- significant path from original IMB model. Regression coefficient are standardized (*P < 0.05, **P < 0.01).

The initial IMB model is shown in Figure 1. However, the model fit statistics for the preliminary model were not acceptable: *χ*^2^ = 195.8, *df* = 15, meaning the *χ*^2^/*df* ratio was not within the acceptable range of 3 or less (*χ*^2^/*df* =13.1). CFI was acceptable at 0.947 but RMSEA was not within the acceptable range of 0.05 or less (RWSEA = 0.148). The initial model thus needed modification.

### Modified IMB model with supplementary paths

**Figure 2:**
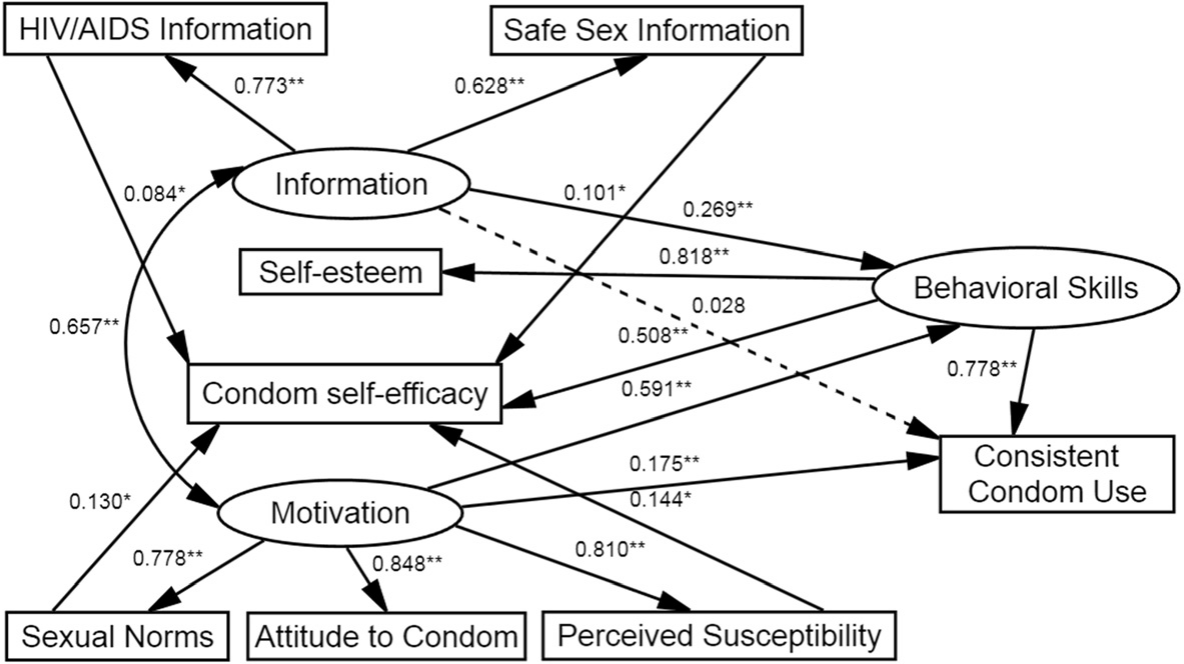
**The final IMB model predicting condom use among 552 senior high school students.** Oval represent multiple-indicator latent variables, rectangle represent single-indicator observable variables. Single-headed arrow represent regression path, double-headed arrows represent correlations. Dotted line indicates non-significant path. Regression coefficient are standardized (*P < 0.05, **P < 0.01).

We modified the IMB model by adding supplementary paths to the initial model. Figure 2 shows the final IMB model. The fit indices for the final model were acceptable after modification: *χ*^2^ = 26.6, *df* = 11, P = 0.139, the *χ*^2^/*df* ratio = 2.42, CFI = 0.981, RMSEA = 0.014. The final IMB model thus showed good fit.

As expected, consistent condom use was strongly predicted by behavioral skills (β = 0.778, *P* < 0.01) and motivation (β = 0.175, *P* < 0.01), but had no significant association with information (β = −0.028, *P* > 0.05). Motivation (β = 0.591, *P* < 0.01) and information (β = 0.269, *P* < 0.01) were significantly and positively associated with behavioral skills, which indirectly affected consistent condom use. Additionally, four new paths were found in the final model, namely that HIV/AIDS information (β = 0.084, *P* < 0.05), safe sex information (β = 0.101, *P* < 0.05), perceived susceptibility to HIV/AIDS (β = 0.144, *P* < 0.05) and sexual norms (β = 0.130, *P* < 0.05) significantly affected condom self-efficacy, a behavioral skill that in turn positively affected consistent condom use.

## Discussion

Adolescents often suffer from sexual and reproductive health problems including HIV/AIDS that result from pre-marital sexual practices [29]. The prevalence of pre-marital sexual intercourse among adolescents ranges from below 5% to over 50% depending on national differences in traditional culture and socioeconomic environment [30-32]. Our research found that 4.5% of senior high school students reported having had premarital sex, which resembles the findings of a national sample of urban adolescents in China conducted in 2010 [14]. Although the prevalence of pre-marital sexual intercourse was lower in our study than in many other countries [31,32], the prevalence of risky sexual behaviors was higher. Only 25% of the sexually active students in our study reported condom use during their first sexual experience, and only about one-ninth reported consistent condom use. The rate of unprotected sexual intercourse among senior high school students in our study exceeds the rates for their peers in other countries, which similar studies found to range from 14.0 to 52.6% [7,9,11,33]. Additionally, the senior high school students displayed poor knowledge and awareness of HIV/AIDS and safe sex. The results suggest an urgent need for STI/AIDS and safe sex related interventions for senior high school students in China.

Consistent condom use seems to be the most effective form of HIV/AIDS prevention among youth. Therefore, it is very important to develop an HIV/AIDS prevention program to change individual risk behavior under the guidance of health promotion theoretical frameworks. One such theoretical framework is the IMB model which has been successfully tested in various HIV/AIDS risk groups [22-24]. Our study also focuses on the ability of the IMB model to predict HIV/AIDS preventive behaviors among senior high school students. The IMB model represents the first attempt to identify the specific linkages among constructs related to HIV/AIDS prevention. The model posits that individuals will maintain HIV/AIDS preventive behavior if they are well-informed, motivated and perceive themselves as possessing the behavioral skills necessary to prevent HIV/AIDS [34]. The IMB model assumes HIV/AIDS prevention information and motivation affect consistent condom use via behavioral skills, while information and motivation exert a direct effect. Consistent with the model assumptions, more information (i.e. higher level of HIV/AIDS and safe sex knowledge), more personal motivation (i.e. positive attitude toward condom use, stronger feeling of vulnerability or susceptibility to HIV/AIDS and a more open attitude to sex), and more preventive skills(i.e. higher condom self-efficacy and self-esteem)were all associated with consistent condom use.

Model fit analysis indicates the initial design of the IMB model applied here is not acceptable and the information is not directly associated with preventive behavior. This suggests the original IMB model should be modified by path analysis. However, the modified model with good fit failed to find a significant association between information and preventive behaviors for senior high school students. Other studies on high HIV/AIDS risk populations have drawn the same conclusion which suggests information is an important but insufficient precursor to HIV-risk prevention behavior [20,26]. However, information may be indirectly associated with preventive behavior via behavioral skills. In the final model, two additional paths show that both HIV/AIDS and safe sex information affect condom self-efficacy. This indicates that participants with high HIV/AIDS and safe sex knowledge are more likely to either practice condom use themselves or require their partners to do so. In the final IMB model, behavioral skills are the most important influence on preventive behavior. This suggests that enhancing condom self-efficacy and self-esteem will directly affect consistent condom use among senior high school students. Meanwhile, motivation is important in directly promoting consistent condom use. It is easy to understand that students with a positive attitude to condom use and high perceived risk of HIV/AIDS are more likely to use condoms consistently. Motivations also contribute indirectly to preventive behavior by affecting behavioral skills. The results suggest that the future intervention should strengthen the motivations and behavioral skills necessary to reduce risky behaviors among senior high school students.

Our study has several limitations. First, data collection is through self-reporting and respondents might misreport their behaviors or attitudes because sex remains a sensitive topic in China. While this bias cannot be determined from the data, reliability and validity studies indicated good test-retest and fit of the results for the questionnaire used [25]. Second, the field test excluded 12th-grade students which might limit generalization from the research findings. Third, we tested a theoretical model by using a structural equation model with specified paths and measured the constructs using cross-sectional data, which can only reveal the associations between constructs observed at a single point in time. Future research should use longitudinal data are needed to observe these same effects.

Overall our study is the first known investigation of which we are aware that examines the utility of the IMB model for predicting consistent condom use among senior high school students in China. The findings highlight the importance of HIV/AIDS health promotion to decrease risk behaviors. The IMB model potentially offers an effective theoretical foundation for HIV/AIDS intervention and suggests that interventions among senior high school students should focus on improving motivations and behavioral skills.

## Conclusions

The prevalence of pre-marital sexual intercourse among senior high school students in China is low when compared with other countries, but that of the risky sexual behaviors is high. Behavioral skills such as condom self-efficacy most significantly influence consistent condom use. Motivations such as attitude to condom use and perceived susceptibility to HIV/AIDS not only contribute to consistent condom use directly but also do so indirectly by affecting behavioral skills. This suggests the importance of decreasing risky sexual behaviors among senior high school students in China and that future interventions should focus on improving motivations and behavioral skills as the final IMB model indicates.

## Abbreviations

HIV/AIDS: Human immunodeficiency virus / acquired immunodeficiency syndrome; IMB: Information-motivation-behavioral skills; SEM: Structural equation modelling; CFI: Comparative fit index; RMSEA: Root mean square error of approximation; CFA: Confirmatory factor analysis.

## Competing interests

The authors declare that they have no competing interests.

## Authors' contributions

All authors contributed the design of this research. YC drafted the manuscript and has been involved in the interpretation of the data. YC and JR performed statistical analyses; XY, RS, GX, LS played a major role in the field survey. YC and HH made a substantial contribution to the interpretation of the data and had been involved in revision of the manuscript through all stages. All authors read and approved the final manuscript.

## Pre-publication history

The pre-publication history for this paper can be accessed here:

http://www.biomedcentral.com/1471-2334/13/262/prepub

## Supplementary Material

Additional file 1 Questionnaire of AIDS health promotion for senior high school students (English version). Click here for file
