# Supplementary material for: Predictors of consistent condom use based on the Information-Motivation-Behavior Skill (IMB) model among senior high school students in three coastal cities in China
Source: BMC Infect Dis. 2013 Jun 4;13:262. doi: 10.1186/1471-2334-13-262 (PMC3686691; doi:10.1186/1471-2334-13-262)
Supplement: Additional file 1 — Questionnaire of AIDS health promotion for senior high school students (English version). [file 1471-2334-13-262-S1.doc]

Questionnaire of AIDS health promotion for senior high school students

**PLEASE READ THE FOLLOWING STATEMENT CAREFULLY BEFORE COMPLETING THE QUESTIONNAIRE.**

**We would like to ask you some questions about yourself, your ideas regarding different aspects of HIV/AIDS prevention to facilitate further health education. This is an anonymous survey for population study. Your answers will be kept confidential and only the researchers and study personnel will have access to this information. Please be as honest and open as possible in your answers.**

**Please mark with an “○” or “√” on your answers after reading each instruction carefully.**

**Many thanks for your patience and participation.**

Jan, 2007

**Section 1: Background Characteristics**

A1. District

A2. School Grade Class

A3. Birthday

A4. What is the highest level of school your father attends?

①primary school ②junior high school ③senior high school (including vocational and technical high school) ④[junior](http://dict.youdao.com/w/junior/) [college](http://dict.youdao.com/w/college/) and above

A5. What is the highest level of school your mother attends?

①primary school ②junior high school ③senior high school (including vocational and technical high school) ④[junior](http://dict.youdao.com/w/junior/) [college](http://dict.youdao.com/w/college/) and above

A6. What is the monthly per capita income of your family?

①below ￥500 ②￥500～999 ③￥1000～1999 ④above ￥2000

**Section 2:** Information

| **B** Reproductive health related knowledge | YES | NO | NOT SURE |
| --- | --- | --- | --- |
| 1. Sperm is produced from the testicle | ① | ② | ③ |
| 2．Ovum is produced from the ovary | ① | ② | ③ |
| 3. Do you think the most risky period for conception is about 3 days before menstruation | ① | ② | ③ |
| 4. The best age to carry a baby is from 24-29 years old | ① | ② | ③ |
| 5. Do you think condom use can prevent pregnancy | ① | ② | ③ |
| 6. Condom should be put on the penis before inserting | ① | ② | ③ |

| **C** HIV/AIDS related knowledge | YES | NO | NOT SURE |
| --- | --- | --- | --- |
| 1. People with multiple sexual partners are more likely to catch HIV/AIDS? | ① | ② | ③ |
| 2. HIV/AIDS can be transmitted from a mother to her child during pregnancy or childbirth? | ① | ② | ③ |
| 3. HIV can be transmitted through sharing a meal. | ① | ② | ③ |
| 4. The HIV incubation period is quite long, one infected with HIV can look perfectly normal without any symptoms. | ① | ② | ③ |
| 5. HIV can be transmitted to the fetus through the placenta. | ① | ② | ③ |
| 6. HIV can be transmitted through sharing toilets and baths.. | ① | ② | ③ |
| 7. HIV can be transmitted through sexual contact, blood, and from mother to the infant. | ① | ② | ③ |
| 8.   HIV can be transmitted through mosquito bites. | ① | ② | ③ |
| 9. HIV can be transmitted through shaking hands. | ① | ② | ③ |
| 10. HIV can be transmitted through sharing a swimming pool. | ① | ② | ③ |
| 11. There is no drug to slow down the development of AIDS so far. | ① | ② | ③ |
| 12. There is no vaccine to prevent HIV/AIDS so far. | ① | ② | ③ |

**Section 3: Motivation**

| D What’s your attitude on the following opinions? | Strongly Disagree | Disagree | Don’t Care | Agree | Strongly Agree |
| --- | --- | --- | --- | --- | --- |
| 1.  You are more likely to get HIV/AIDS if you have no knowledge of this disease? | ① | ② | ③ | ④ | ⑤ |
| 2. HIV/AIDS epidemic is rapidly growing. | ① | ② | ③ | ④ | ⑤ |
| 3. Condoms should be used when having sex? | ① | ② | ③ | ④ | ⑤ |
| 4. Sex without a condom should be refused? | ① | ② | ③ | ④ | ⑤ |
| 5. One can reduce the risk of HIV infection by using condom. | ① | ② | ③ | ④ | ⑤ |
| 6．One can reduce the risk of accidental pregnancy by using condom. | ① | ② | ③ | ④ | ⑤ |
| 7. Do you agree with a girl having premarital sex with her boyfriend? | ① | ② | ③ | ④ | ⑤ |
| 8．Do you agree with a boy having premarital sex with his girlfriend? | ① | ② | ③ | ④ | ⑤ |
| 9. Do you agree with a man to have extramarital affairs? | ① | ② | ③ | ④ | ⑤ |
| 10. Do you agree with a woman to extramarital affairs? | ① | ② | ③ | ④ | ⑤ |

**Section 4: Behavioral Skill**

| E Self-esteem Scale | Strongly Disagree | Disagree | Agree | Strongly Agree |
| --- | --- | --- | --- | --- |
| 1. I am able to do things as well as most other people | ① | ② | ③ | ④ |
| 1. I have many good characteristics. | ① | ② | ③ | ④ |
| 1. I think I’m a loser. | ① | ② | ③ | ④ |
| 1. I am at least as valuable as others. | ① | ② | ③ | ④ |
| 1. I have nothing to be proud of. | ① | ② | ③ | ④ |
| 1. I feel positive about myself. | ① | ② | ③ | ④ |
| 1. On the whole， I’m satisfied with myself. | ① | ② | ③ | ④ |
| 1. I should think highly of myself. | ① | ② | ③ | ④ |
| 1. Sometimes I think I’m useless. | ① | ② | ③ | ④ |
| 1. I think I’m good for nothing. | ① | ② | ③ | ④ |

F1. I can use a condom during sexual intercourse?

1. Absolutely Not ②Probably Not ③Probably Yes ④Absolutely Yes

F2. I can persuade my partner to use a condom during sexual intercourse?

1. Absolutely Not ②Probably Not ③Probably Yes ④Absolutely Yes

**Section 5: Behavior**

G1. Have you been in love with someone? ①Yes ②No

G2. Have you done the following things so far?

1.Kiss ①Yes ②No

2.Hug ①Yes ②No

3.Foreplay ①Yes ②No

4.Sexual intercourse ①Yes ②No（to the end）

5. Did you use condom during sex? ①Yes ②No（to the end）

6. Did you use condom consistently during your sexual intercourse in the past 6 months?

①Never ②Occasionally ③Sometimes ④Usually ⑤Always
